# Supplementary material for: Analog Memristive Characteristics of Square Shaped Lanthanum Oxide Nanoplates Layered Device
Source: Nanomaterials (Basel). 2021 Feb 9;11(2):441. doi: 10.3390/nano11020441 (PMC7915431; doi:10.3390/nano11020441)
Supplement: Supplementary file 1 [file nanomaterials-11-00441-s001.pdf]

Supporting Information

Analog memristive characteristics of square shaped lanthanum oxide nanoplates layered device

Wonkyu Kang [1], Kyoungmin Woo [1], Hyon Bin Na [1], Chi Jung Kang [2], Tae-Sik Yoon [3], Kyung Min Kim [4] and Hyun Ho Lee [1,*]

1 Department of Chemical Engineering, Myongji University, Gyeonggi-Do Yongin-Si 17058, South Korea; kangwk7@naver.com (W.L.); mywkm1@naver.com (K.W.); hyonbin@mju.ac.kr (H.N.N.)
2 Department of Physics Myongji University, Gyeonggi-Do Yongin-Si 17058, South Korea; cjkang@mju.ac.kr
3 Department of Materials Sciences and Engineering Myongji University, Gyeonggi-Do Yongin-Si 17058, South Korea; tsyoon@mju.ac.kr
4 Department of Materials Sciences and Engineering, KAIST, Daejeon-Si 34141, South Korea; km.kim@kaist.ac.kr
* Correspondence: hyunho@mju.ac.kr

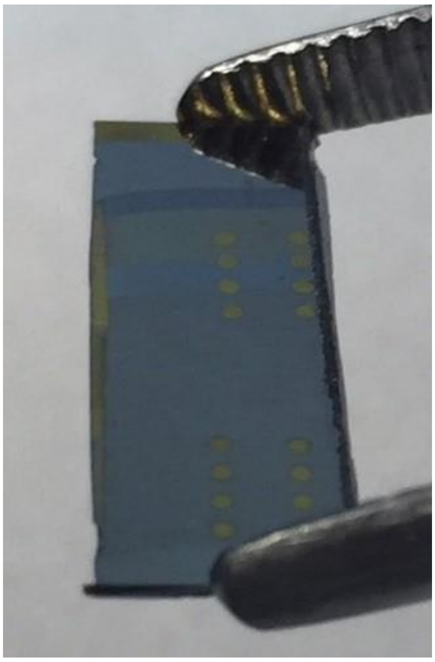

(a)

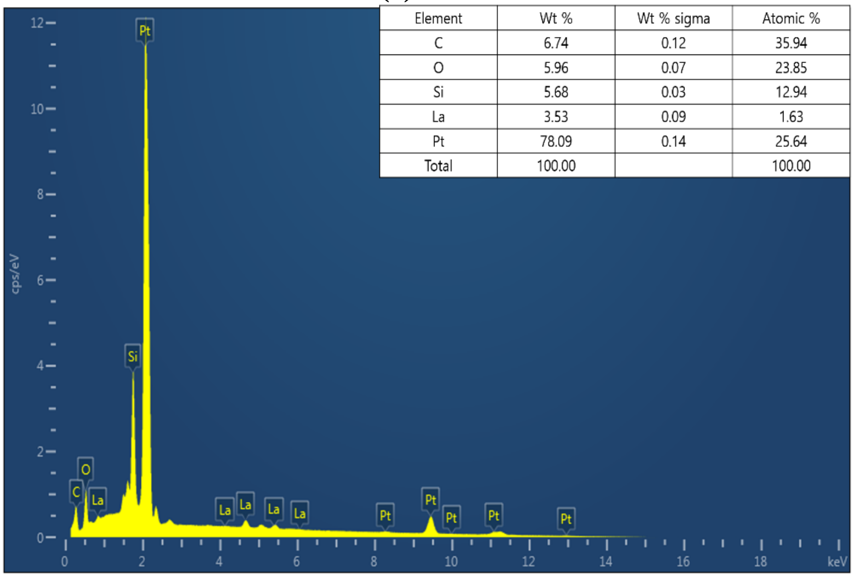

Element	Wt %	Wt % sigma	Atomic %
C	6.74	0.12	35.94
O	5.96	0.07	23.85
Si	5.68	0.03	12.94
La	3.53	0.09	1.63
Pt	78.09	0.14	25.64
Total	100.00		100.00

(b)

Figure S1. (**a**) Photo image of a complete LaO_x NPs layered device having Au top electrode, and (**b**) EDS analysis of atomic ratio on surface of LaO_x NPs layer.

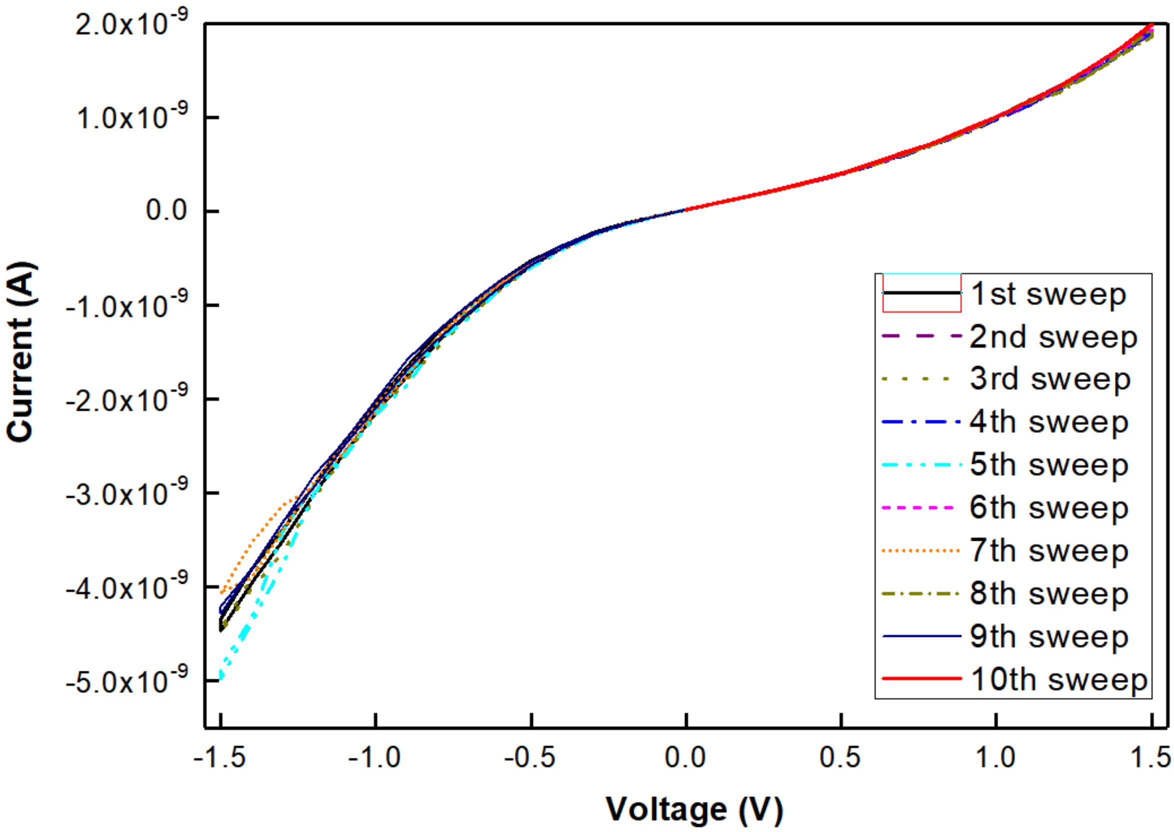

Figure S2. I-V characteristics of ten consecutive sweep of Al/ LaO_x NPs/Pt device

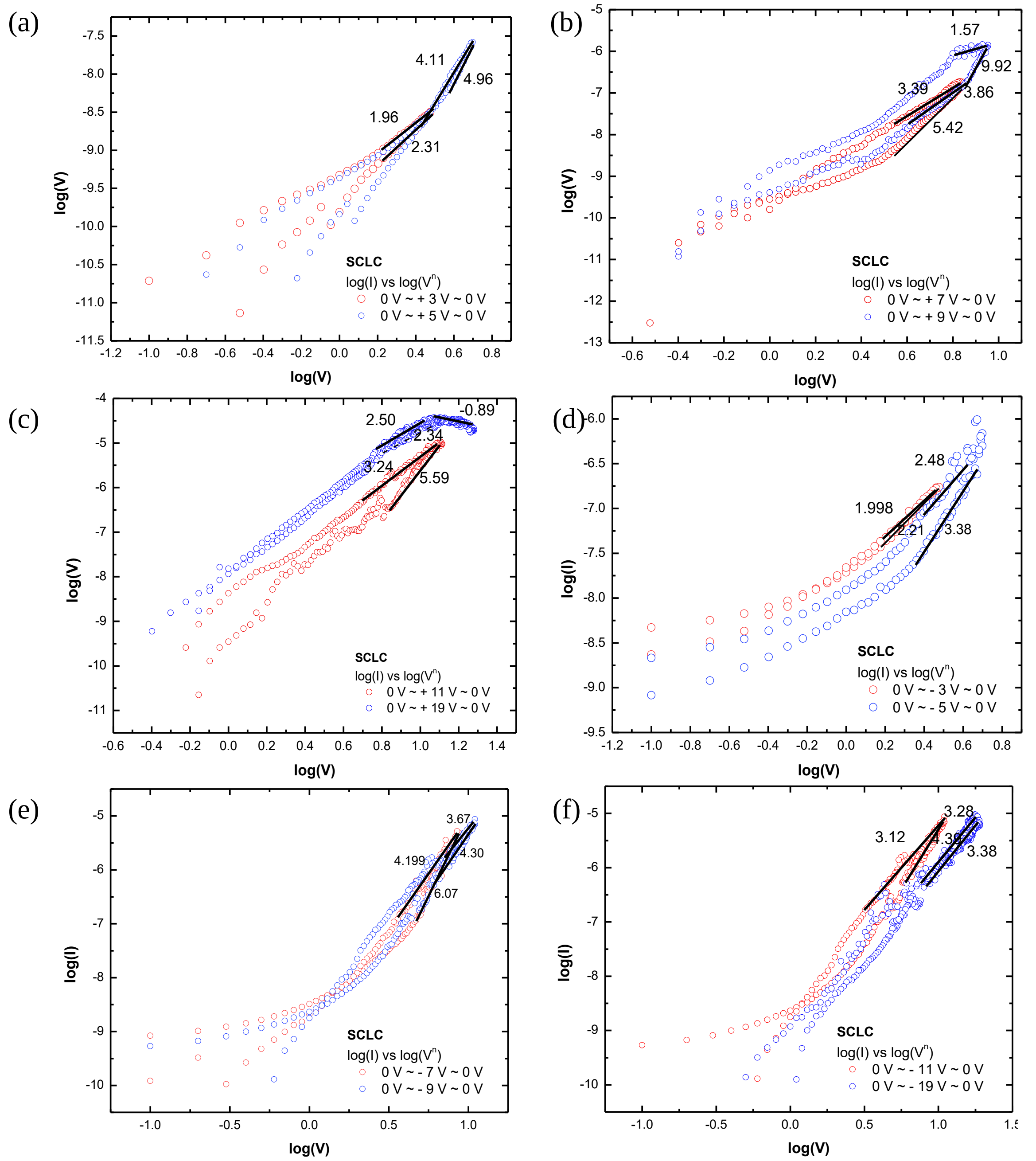

Figure S3. Replot of I - V in Figure 3c with the form of SCLC at (**a**) + 3 V, + 5 sweep, (**b**) –3 V sweep, -5 V sweep, (**c**) + 7, + 9 V sweep, (**d**) – 7 V, - 9 V sweep, (**e**) + 11 V sweep, +19 V sweep and (**f**) -11 V sweep, -19 V sweep.
